# The interplay between structural flood protection, population density, and flood mortality along the Jamuna River, Bangladesh

**DOI:** 10.1007/s10113-020-01600-1

**Published:** 2020-02-03

**Authors:** Md Ruknul Ferdous, Giuliano Di Baldassarre, Luigia Brandimarte, Anna Wesselink

**Affiliations:** 1 Faculty of Social and Behavioural Sciences, University of Amsterdam, 1012 WX Amsterdam, The Netherlands; 2 Department of Integrated Water Systems and Governance, IHE Delft Institute for Water Education, 2611 AX Delft, The Netherlands; 3 Department of Earth Sciences, Uppsala University, SE-75236 Uppsala, Sweden; 4 Centre of Natural Hazards and Disaster Science, CNDS, SE-75236 Uppsala, Sweden; 5 Department of Sustainable Development, Environmental Sciences and Engineering, KTH, SE-10044 Stockholm, Sweden

**Keywords:** Flood risk management, Socio-hydrology;, Levee effect;, Bangladesh

## Abstract

Levees protect floodplain areas from frequent flooding, but they can paradoxically contribute to more severe flood losses. The construction or reinforcement of levees can attract more assets and people in flood-prone area, thereby increasing the potential flood damage when levees eventually fail. Moreover, structural protection measures can generate a sense of complacency, which can reduce preparedness, thereby increasing flood mortality rates. We explore these phenomena in the Jamuna River floodplain in Bangladesh. In this study area, different levels of flood protection have co-existed alongside each other since the 1960s, with a levee being constructed only on the right bank and its maintenance being assured only in certain places. Primary and secondary data on population density, human settlements, and flood fatalities were collected to carry out a comparative analysis of two urban areas and two rural areas with different flood protection levels. We found that the higher the level of flood protection, the higher the increase of population density over the past decades as well as the number of assets exposed to flooding. Our results also show that flood mortality rates associated with the 2017 flooding in Bangladesh were lower in the areas with lower protection level. This empirical analysis of the unintended consequences of structural flood protection is relevant for the making of sustainable policies of disaster risk reduction and adaptation to climate change in rapidly changing environments.

## Introduction

In the year 1964, new levees were built to prevent flooding in the village of Char Jabbar, Bangladesh. The presence of this structural protection measure encouraged more human settlements and numerous people moved into this flood-protected area (Burton et al. 1993). A few years later, in November 1970, a tropical cyclone hit Bangladesh, levees were overtopped, and about 6000 people were killed by flooding (Islam 1971). The dramatic history of Char Jabbar shows how the net effect of building levees can result into increasing flood losses and fatalities (White 1945).

Char Jabbar is not an exceptional case. Over the past decades, numerous scholars have shown that structural flood protection tends to be associated with increasing flood exposure, defined here as the population and assets located in flood hazard-prone areas (Jongman et al. 2015), and flood vulnerability, defined here as the susceptibility of the exposed elements to flooding (Jongman et al. 2015). This tendency is typically described as the ‘safe development paradox’, ‘levee effect’, ‘residual risk’, or ‘safety dilemma’ and it was shown to potentially offset the intended benefits of structural flood protection (e.g., White 1945; Tobin 1995; Kates et al. 2006; Burby 2006; Montz and Tobin 2008; Scolobig and De Marchi 2009; Di Baldassarre et al. 2013a, b).

Several studies have shown that increasing the levels of structural flood protection can attract more settlements and high-value assets in the protected areas (e.g., White 1945; Kates et al. 2006; Montz and Tobin 2008; Di Baldassarre et al. 2013a, b), thereby increasing exposure to flooding. Kates et al. (2006), for example, discussed that the catastrophic 2005 flooding of the New Orleans (Katrina) showed that while flood defense has reduced the negative consequences associate with more frequent events, it also contributed to build up exposure to more rare events. Other studies have explored how structural flood defense can generate a sense of complacency (Tobin 1995), which can act to reduce preparedness, thereby increasing social vulnerability to flooding (e.g., Burby 2006; Scolobig and De Marchi 2009; Ludy and Kondolf 2012). For instance, Ludy and Kondolf (2012) looked at Sacramento-San Joaquin Delta where the residual risk for lands protected by a 200-year levee is extremely high but it is completed ignored (or vastly underestimated) by locals. Literature in this field is vast, as shown in the recent review made by Di Baldassarre et al. (2018), and it goes well beyond river and coastal flooding. Logan et al. (2018), for example, analyzed tsunami impacts in Taro, Japan. They observed that structural protection measures can cause a false sense of security and encourage development that is vulnerable in the long-term.

The safe development paradox should not be seen as a mere one-way causal link, but the result of self-reinforcing (bidirectional) feedbacks (Di Baldassarre et al. 2013a, 2013b): e.g., increasing protection levels enable intense urbanization that will in turn plausibly require even higher protection standards (Viglione et al. 2014). Thus, it can generate the lock-in conditions towards exceptionally high levels of flood protection and extremely urbanized floodplains (Di Baldassarre et al. 2018). This lock-in condition can become unsustainable (e.g., maintenance costs) or socially unjust, as the costs and benefits of flood protection measures, as well as potential flood losses, are not always fairly shared across social groups (Burton and Cutter 2008), as seen for instance in the aftermath of the catastrophic 2005 flooding of New Orleans (Masozera et al. 2007).

The recent literature has not only shown how building or raising levees can lead to very intense occupation (with more people and assets than originally expected) of flood-prone areas behind the levee but also losses of ecological functions (Opperman et al. 2009). Yet, numerous structural protection structures, such as levees or flood-control reservoirs, are being suggested, planned, or built in many areas around the world, as the narrative that “we need to building higher levees to cope with flooding” remains pervasive not only for policy and decision makers but also within the scientific community (Ward et al. 2017; Di Baldassarre et al. 2018).

Kreibich et al. (2017) explains the reduction of mortality rates in Bangladesh as a result of different factors, including early warning systems based on better flood forecasting (Gain et al. 2015) along with more spontaneous or informal processes, such as the combination of higher education and flood experience leading to increased awareness and preparedness.

This tendency of decreasing flood losses over time is termed ‘adaptation effect’ in the literature (Di Baldassarre et al. 2015) and it has been observed by other studies (Jongman et al. 2015; Mechler and Bouwer 2015; Kreibich et al. 2017) across different socio-hydrological contexts. Yet, the literature has also shown that adaptation effects are less significant when the levels of structural flood protection are very high, due to higher reliance (and trust) on levees or flood-control reservoirs (Mård et al. 2018). As such, one of the questions guiding our research work is: how are flood mortality rates and people settled in flood-prone areas influenced by structural flood protection in Bangladesh?

**Fig. 1:**
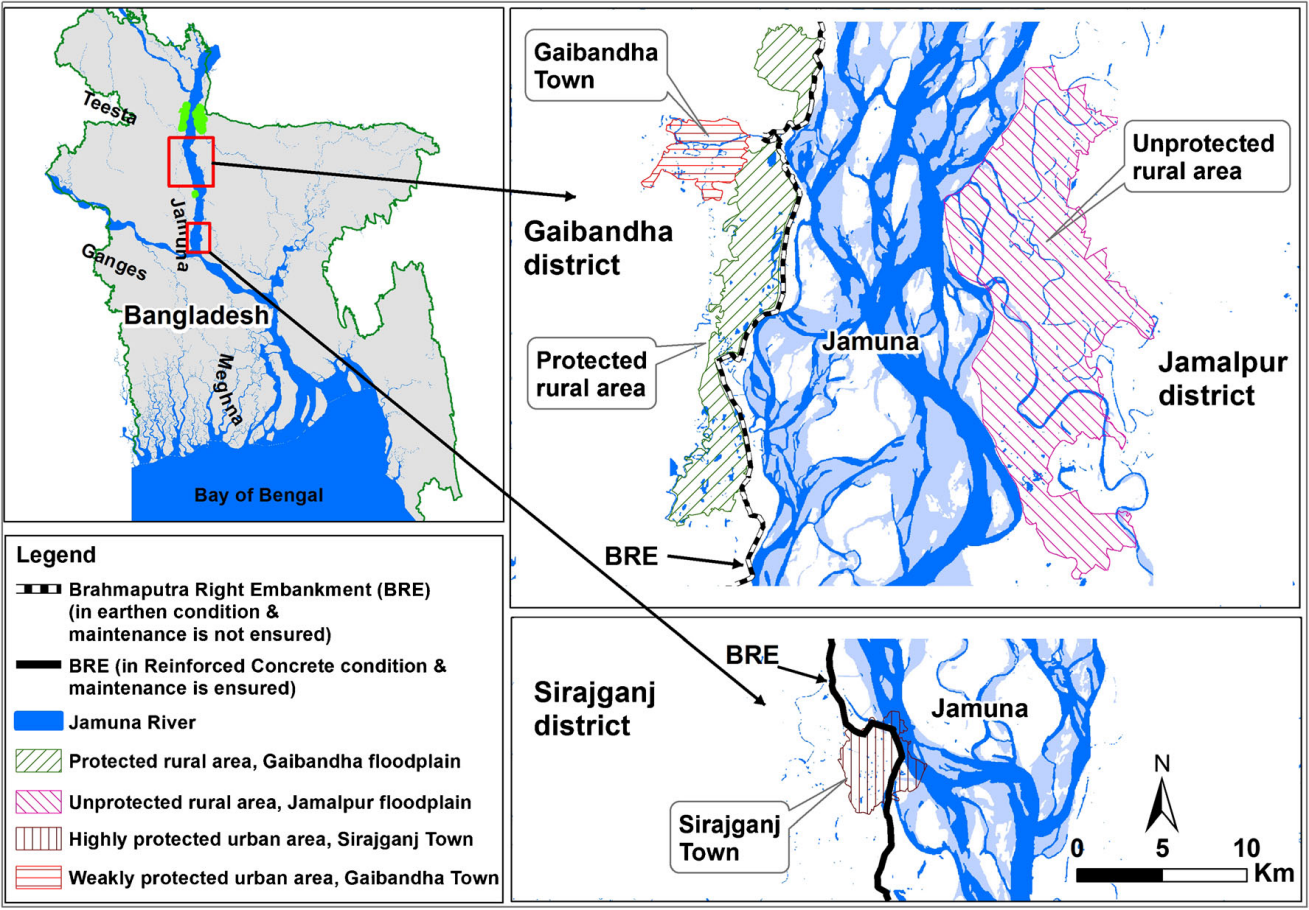
Bangladesh and its major rivers. The two insets shows the Brahmaputra Right Embankment (BRE) and the four study areas: the protected rural area in the Gaibandha district, the unprotected rural area in the Jamalpur district, and two urban areas (Gaibandha Town and Sirajganj Town) with different flood protection levels

To address this question, we explore different socio-hydrological spaces (Ferdous et al. 2018) in the Jamuna River floodplain in Bangladesh (Fig. 1). This study area is different as levels of flood protection have co-existed alongside each other since the 1960s, with a levee (i.e., the Brahmaputra Right Embankment, BRE) being constructed only on the right bank and its maintenance being assured only in certain places (Fig. 1). This consists of four test sites characterized by different levels of structural flood protection (Fig. 1). Primary and secondary data on population density, human settlements, and flood fatalities were collected to carry out a comparative analysis of two urban areas and two rural areas with different flood protection levels.

## Study area

In the year 2017, major flooding hit Bangladesh. Almost half (42%) of the country is under water (FFWC/BWDB 2018), and in numerous island villages along the Jamuna River, entire homes have been washed away, while crops and food supplies all but wiped out. “Villagers described the rains as the worst in living memory” (CNN 2017). According to FFWC/BWDB (2018), the 2017 flood hit the country twice: on 1st week of July and on 2nd week of August due to excessive rainfall in the upstream of Bangladesh. In both cases, flood duration was about 2 weeks, but the second flood peak in August was more severe. The water level of the Jamuna River crossed the danger level on around 3rd week of August and remained above it for about 1 month. In the previous 100 years, the highest water level of Jamuna River was the one recorded 20.62 m PWD (Public Work Department, i.e., above mean sea level) at Bahadurabad station in 1988, but such highest water level was exceeded to 20.84 m PWD at the same station in 2017 thereby setting a new flood peak record (FFWC/BWDB 2018).

**Fig. 2:**
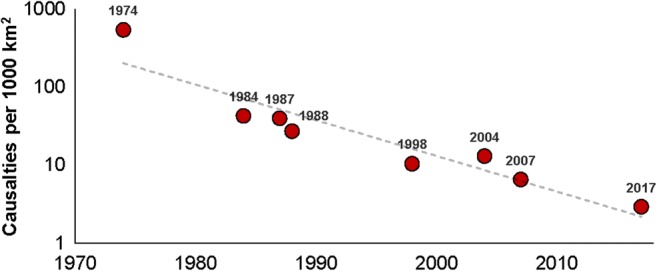
Flood fatalities in Bangladesh normalized by flooded area (casualties by 1000 km^2^) for major flooding events between 1974 and 2017. (Data source: Brammer (2004); Sultana et al. (2008); Penning-Rowsell et al. (2012); BBS (2016a, b); NDRCC (2017))

Indeed, data of flood losses (about 0.7 million houses and crops of about 0.6 million hectares land were damaged) and fatalities (recorded total is 147) shows that the negative impacts of the 2017 flooding in Bangladesh were massive. Yet, when compared to the most recent events (Fig. 2), one can observe that flood mortality rates in Bangladesh have been significantly decreasing over time, as previously observed by Mechler and Bouwer (2015).

To better understand the interplay between structural protection levels and flood exposure/mortality, we explore the effects of structural flood protection in four different types of human settlements along the Jamuna River floodplain. They consist of two rural environments – the protected rural area in the Gaibandha district (an embankment was constructed in the 1960s parallel to the west bank of the Jamuna River to restrict flood water to enter in that area) and the unprotected rural area (no man-made embankment was constructed along the east bank to restrict flood water) in the Jamalpur district – and two urban environments, Gaibandha and Sirajganj, with different levels of structural flood protection (Fig. 1 and Table 1).

**Table 1:** Summary of socio-economic factors in the four study areas (BBS 2013). Chi-Square test at 5% level shows that disabled population, sex ratio and age are not significantly different, while people with electricity facility and literacy rates are significantly different

	Urban, more protected Sirajganj town	Urban, less protected Gaibandha town	Rural, protected Gaibandha floodplain	Rural, unprotected Jamalpur floodplain
Flood early warning system?	Yes	Yes	Yes	Yes
Disabled population	1.5%	1.6%	1.5%	1.4%
Sex ratio	1.02	1.00	1.00	0.97
Literacy Rate	63.2%	74.5%	31.2%	30.6%
Average age	26.7 years	29.5 years	25.0 years	25.2 years
People with electricity facility	90.1%	84.8%	41.8%	21.0%

The protected rural area in the Gaibandha district (right bank of the Jamuna river, Fig. 1) has a total surface of about 74 km^2^ and a population of approximately 111,000 people (BBS 2013). This rural area is protected by regular annual flooding which is locally termed as normal flooding (Ferdous et al. 2018). However, a few locations of this area are still frequently inundated by excessive rainfall or adjacent small rivers (Alai and Ghagot).

The unprotected rural area in the Jamalpur district (left bank of the Jamuna river, Fig. 1) has a total surface of about 174 km^2^ and a population of approximately 146,000 people (BBS 2013). As there is no man-made structural protection measure in this rural area, flooding occurs more frequently here than on the right bank. Some other small rivers (e.g., Old Brahmaputra and Jinjira) flow adjacent to this area and contribute to flooding in this area.

The Gaibandha town area has a total surface about 17 km^2^ and a population of approximately 68,000 people (BBS 2013). This urban area is protected from normal floods with a relatively weak levee system that consists of the BRE and other two embankments constructed in 1995 along two small tributaries (i.e., Ghagot and Alai) of the Jamuna River. The BRE effectively protects the area against frequent flooding from the Jamuna and, as a result, inhabitants feel relatively confident to invest in businesses and homesteads (Rahman 2017; Ferdous et al. 2018). The last extreme flooding events in Gaibandha occurred in 1988 and 2017.

The Sirajganj town area has a total surface about 19 km^2^ and a population of approximately 160,000 people (BBS 2013). This urban area is protected from flooding with a relatively stronger levee system, as the BRE was heightened and reinforced in the 1990s to protect this town from frequent flooding. Still, flooding occurred both in 1988 and 2017. The BRE effectively protects the area against most flooding events from the Jamuna and, as a result, inhabitants feel relatively confident enough to invest in businesses and homesteads (Rahman 2017).

## Data and methods

Our study builds upon previous work about flood risk in Bangladesh (Haque and Zaman 1993; Brammer 2010; Cook and Lane 2010; Cook and Wisner 2010; Mechler and Bouwer 2015; Gain et al. 2015; Ferdous et al. 2018). Our analysis is based on secondary data for two urban and two rural areas with different protection levels. In these four study areas, we collected secondary data on population density, satellite images (for human settlements), and flood fatalities and carry out a comparative analysis about the effects of structural flood protection. Time series of the national census from 1974 to 2011 were provided by the Bangladesh Bureau of Statistics (BBS) and from 1901 to 1961 were provided by Census of Pakistan Population (CPP) (Table 2). We used this data to see the increase in population densities in the four study areas. Flood fatalities data were collected from National Disaster Response Coordination Centre (NDRCC) of the Government of Bangladesh (Table 2) to analyze the trend of flood fatalities over time. Time series of satellite images of 30 m spatial resolution were provided by CEGIS, Bangladesh (Table 2). These datasets were used to analyze the expansion of human settlements in the four study areas. Land use and land cover classifications were carried out using optical images with high spectral resolution (7 bands for Landsat 4/5 and 11 bands for Landsat 8). Due to lack of availability of remote sensing data with high spatial resolution prior to 1989, we analyzed land use patterns only for the years 1989 and 2014, thereby showing expansion of the human settlement areas over the last three decades. These were also overlaid to the 2017 flood extent map provided by the Dartmouth Flood Observatory (Brakenridge 2019) in order to explore the proportions of territory that was flooded in protected and unprotected areas. All images were geo-rectified into “Bangladesh Transverse Mercator” (BTM) projection. For better visual interpretation, the false-color composition was used. After visual interpretation, 50 spectral classes were generated using a digital unsupervised classification to derive different land uses and land covers from the satellite images. ERDAS IMAGINE software uses the ISODATA, stands for “Iterative Self-Organizing Data Analysis Technique”, algorithm to perform this classification. The ISODATA clustering method uses the minimum spectral distance formula to form clusters. After digital classification, the mixed classes were grouped together, and the similar process was run for refining the classes and increasing accuracy level. The 2014 settlement data were taken from vector data, digitized from multispectral RapidEye (5 m of spatial resolution) images. These vector data were converted into raster format with the software ERDAS IMAGINE and used for land use classification.

**Table 2:** Summary of data used for the spatial analysis

**Parameter**	**Years**	**Information**	**Source**
Satellite images - Landsat 4 TM - Landsat 8	1989 2014	- Spatial resolution 30 m - Spectral resolution 7 bands - Spatial resolution 30 m - Spectral resolution 11 bands	CEGIS
Population	1901–2011		CPP (1964); BBS (1974); BBS (1986); BBS (1994); BBS (2005); BBS (2013); BBS (2014a); BBS (2014b)
Flood fatalities	1974–2017		Brammer (2004); Sultana et al. (2008); Penning-Rowsell et al. (2012); BBS (2016a, b); NDRCC (2017)

## Results

**Fig. 3:**
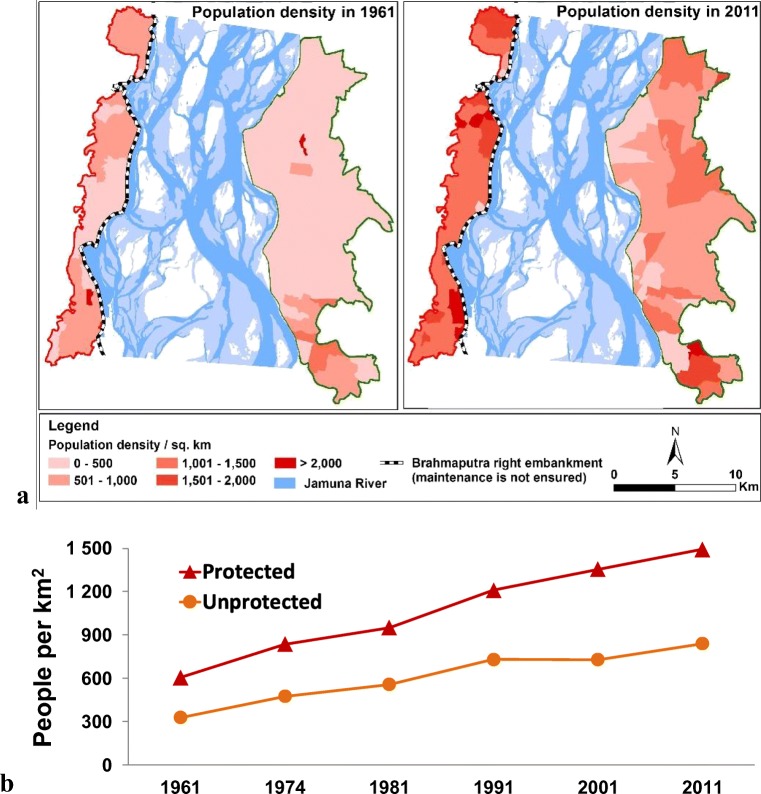
Flood exposure in rural areas: **a**) Population density in 1961 and 2011. **b**) Population density in the period 1961–2011

Spatial and temporal changes in flood exposure in the two rural areas are depicted in Fig. 3. In particular, Fig. 3a shows the spatial distribution of population density in 1961 and 2011, while Fig. 3b shows density in the period 1961–2011. While both protected and unprotected areas have been increasing since 1961, the results of our study show that protected areas have had a large increase of population density.

**Fig. 4:**
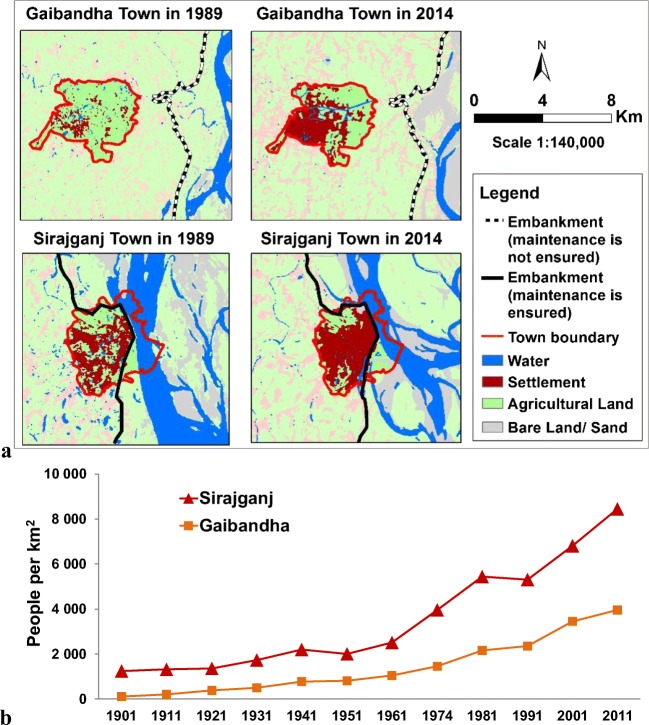
Flood exposure in urban areas: **a**) Land use patterns in 1989 and 2014. **b**) Population density in the period 1901–2011

Urban areas are compared in Fig. 4, which depicts the temporal and spatial evolution of urbanization patterns between 1989 and 2014 (Fig. 4a) and population density (Fig. 4b) in the period 1901–2011. Before the construction of the BRE, both urban areas show moderate increase of population, with a similar rate of growth. After the construction of the BRE, both urban areas show a change in the population growth rate: Sirajgani shows a much steeper increase than Gaibandha. The severe effects of major floods occurred in 1987 and 1988 are visible in a drop of population growth between the year 1981 and 1991. These outcomes show that after the reinforcement of the levee system in Sirajgani, the town has had more growth in human population than in Gaibandha.

To corroborate these findings, we also compared changes in population density over the past 30 years in Shahjadpur Upazaila on the west bank of Jamuna river (protected) and in Nagarpur Upazila on the east bank of Jamuna river (unprotected). We found that population density is higher in the protected area (1730 persons/km^2^ vs. 1100 persons/km^2^). Also the increase rate in population density is higher in the protected area (22 persons/km^2^/year vs. 10 persons/km^2^/year).

Moreover, the analysis of flood fatalities caused by the 2017 flooding for our study area showed that the flood mortality rates in the areas with lower protection level were less than the flood mortality rates in the areas with higher protection levels: 1 vs. 3 fatalities per 100,000 people in unprotected vs. protected rural areas, and 1 vs. 2 per 100,000 people in less vs. more protected urban areas (Source: National disaster response co-ordination centre (NDRCC), Government of Bangladesh). This difference cannot be explained by different levels of exposure since our spatial analysis, based on the 2017 flood extent map provided by the Dartmouth Flood Observatory (Brakenridge 2019), showed that the proportions of territory that was flooded in protected and unprotected areas were very similar (59% vs. 55%, respectively).

Secondary data for socio-economic factors are limited for our study area. We select few demographic and socio-economic factors that might influence the population density in our study area. Table 1 shows that: **i**) flood early warning systems are in place in all four test sites, while sex ratio, age, and the proportion of disabled people are relatively homogeneous.

## Discussion

Our findings show that the prevention of small flooding events via structural measures has not only been associated with more intense urbanization of flood-prone areas (Figs. 3 and 4) but also with higher mortality rates when extreme flooding events eventually occur. We attribute these results to the safe-development paradox (White 1945; Kates et al. 2006; Di Baldassarre et al. 2018). More protected areas attract more assets and people, thereby increasing the potential flood damage when levees eventually fail. Moreover, structural protection measures can generate a sense of complacency, which can reduce preparedness, thereby increasing flood mortality rates. Yet, it should be noted that differences in mortality rates are limited. Moreover, there remain other factors, such as literacy rate, that differ across the four test sites (Table 1) and have unknown effects on flood mortality. As such, our empirical results should be used with caution.

The results of this case study support consolidated theories about the interplay between levels of structural flood protection, people and assets exposed to flooding, and social vulnerability to flooding. While similar outcomes have been broadly discussed in the flood risk literature with reference to US, European, and Australian cases studies (e.g., Tobin 1995; Kates et al. 2006; Di Baldassarre et al. 2018), this is the first study providing empirical evidence of these phenomena in a low-income country. Moreover, the presence of four adjacent study areas with different protection standards enabled an original comparative analysis. As such, the results of this study are relevant for the making of sustainable policies of flood risk reduction and adaptation to climate change in Bangladesh, and inform socio-hydrological models integrating human behavior in risk analysis (e.g., Sivapalan et al. 2012; Di Baldassarre et al. 2015; Aerts et al. 2018). For instance, we found that more protected areas experience higher flood losses during severe flooding events, but these areas experience less year-by-year damage caused by ordinary floods. Moreover, they have had relatively more economic growth (e.g., access to electricity; Table 1), investments, and agricultural incomes (Ferdous et al. 2019). These outcomes can be used to parameterize conceptual models of human-flood interactions (Di Baldassarre et al. 2015) as well as risk assessment methods (Aerts et al. 2018).

Blöschl et al. (2013) distinguish between a top-down rationale for flood risk management, where decisions are based on probabilities of flooding and risk calculation (e.g., cost-benefit analysis), and a bottom-up rationale where the possibility of flooding, social vulnerability and the ability of populations to recover are key for decisions. Our work has unraveled new aspects that can contribute to advance both perspectives in Bangladesh, as the influence of structural flood protection in the historical change of human settlements can improve methods for risk calculation, while the outcomes about mortality rates provide new insights about the link between flood occurrences, preparedness, and coping capacities.

Our study has a number of limitations. The dynamics of human settlements in the Jamuna floodplain are only partly attributable to the combination of the factors presented here, i.e., frequency of flooding events, structural flood protection, and household coping capacities. As a matter of fact, other external factors, such as migration or lack of alternative settlement locations, may have played an important role in shaping the evolution of the four human settlements analyzed here (e.g., Penning-Rowsell et al. 2012; Di Baldassarre et al. 2018). As such, more empirical research is needed how endogenous and exogenous factors shape the dynamics of human settlements and contribute to flood risk changes in Bangladesh. Moreover, while our analysis of flood exposure considers the proportion of the each study area that was flooded in 2017, it does not account for the spatial distribution of population within each study area. As population distribution within each territory is not homogenous in space, this limits the insights about actual flood exposure (e.g., Smith et al. 2019).

## Conclusions

A shift from hard (fighting floods) to soft (living with floods) approaches for flood risk management is a general trend in policy and scientific writing today (e.g., Opperman et al. 2009). In terms of policy implications for flood risk management, various scholars have already argued that Bangladesh should not implement hard engineering work and high levels of structural flood protection, but stick to their traditional softer approach (e.g., Haque and Zaman 1993; Cook and Lane 2010). In fact, some of the polders that were constructed in 1970s, which had negative impacts on livelihoods and ecosystems, are now being partially removed or revised to re-establish a workable sediment and water balance.

Our work contributes to advance the knowledge underpinning flood risk management in Bangladesh. Yet, there are no clear-cut answers to the question of how should Bangladesh cope with flooding in the coming decades because of the aforementioned complexity of endogenous and exogenous factors. Moreover, the balance between soft and hard approaches also depends on the (unavoidably subjective and different) weights and values given by local people, experts, researchers, and governments to economic, environmental, and social benefits and costs. There are, in fact, multiple feasible (and desirable) trade-offs between hard and soft approaches and their identification calls for a transparent communication of positive (often intended) and negative (often unintended) effects of alternative measures in flood risk management.
